# Function of hematopoiesis and bone marrow niche in inflammation and non-hematopoietic diseases

**DOI:** 10.1093/lifemedi/lnaf015

**Published:** 2025-03-26

**Authors:** Yu-xiang Wang, Zhao-hua Deng, Yu-yan Li, Ke Bai, Jinjin Ma, Yang Liu, Qi Chen

**Affiliations:** Center for Cell Lineage Atlas, Joint School of Life Sciences, Guangzhou Institutes of Biomedicine and Health, Chinese Academy of Sciences, Guangzhou Medical University, Guangzhou 510530, China; University of Chinese Academy of Sciences, Beijing 101408, China; China-New Zealand Belt and Road Joint Laboratory on Biomedicine and Health, Guangdong Provincial Key Laboratory for Stem Cell and Regenerative Medicine, Guangdong-Hong Kong Joint Laboratory for Stem Cell and Regenerative Medicine, Joint School of Life Sciences, Guangzhou Institutes of Biomedicine and Health, Chinese Academy of Sciences, Guangzhou Medical University, Guangzhou 510530, China; Center for Cell Lineage Atlas, Joint School of Life Sciences, Guangzhou Institutes of Biomedicine and Health, Chinese Academy of Sciences, Guangzhou Medical University, Guangzhou 510530, China; University of Chinese Academy of Sciences, Beijing 101408, China; China-New Zealand Belt and Road Joint Laboratory on Biomedicine and Health, Guangdong Provincial Key Laboratory for Stem Cell and Regenerative Medicine, Guangdong-Hong Kong Joint Laboratory for Stem Cell and Regenerative Medicine, Joint School of Life Sciences, Guangzhou Institutes of Biomedicine and Health, Chinese Academy of Sciences, Guangzhou Medical University, Guangzhou 510530, China; Center for Cell Lineage Atlas, Joint School of Life Sciences, Guangzhou Institutes of Biomedicine and Health, Chinese Academy of Sciences, Guangzhou Medical University, Guangzhou 510530, China; University of Chinese Academy of Sciences, Beijing 101408, China; China-New Zealand Belt and Road Joint Laboratory on Biomedicine and Health, Guangdong Provincial Key Laboratory for Stem Cell and Regenerative Medicine, Guangdong-Hong Kong Joint Laboratory for Stem Cell and Regenerative Medicine, Joint School of Life Sciences, Guangzhou Institutes of Biomedicine and Health, Chinese Academy of Sciences, Guangzhou Medical University, Guangzhou 510530, China; The Innovation Centre of Ministry of Education for Development and Diseases, School of Medicine, South China University of Technology, Guangzhou 510006, China; The Innovation Centre of Ministry of Education for Development and Diseases, School of Medicine, South China University of Technology, Guangzhou 510006, China; The Institute of Future Health, South China University of Technology, Guangzhou International Campus, Guangzhou 511442, China; The Innovation Centre of Ministry of Education for Development and Diseases, School of Medicine, South China University of Technology, Guangzhou 510006, China; Center for Cell Lineage Atlas, Joint School of Life Sciences, Guangzhou Institutes of Biomedicine and Health, Chinese Academy of Sciences, Guangzhou Medical University, Guangzhou 510530, China; China-New Zealand Belt and Road Joint Laboratory on Biomedicine and Health, Guangdong Provincial Key Laboratory for Stem Cell and Regenerative Medicine, Guangdong-Hong Kong Joint Laboratory for Stem Cell and Regenerative Medicine, Joint School of Life Sciences, Guangzhou Institutes of Biomedicine and Health, Chinese Academy of Sciences, Guangzhou Medical University, Guangzhou 510530, China

**Keywords:** hematopoiesis, bone marrow niche, hematopoietic stem and progenitor cell, inflammation

## Abstract

Hematopoiesis and the behavior of hematopoietic stem and progenitor cells (HSPCs) are regulated by the bone marrow niche. Here, we introduce the major niche cell types in bone marrow and their response to stress condition. We highlight the hematopoietic response and bone marrow niche adaptation to inflammatory condition and non-hematopoietic diseases, which are not systematically summarized. These emerging data suggest targeting hematopoiesis and bone marrow niche may provide novel therapeutic target to precisely control the progression of the diseases.

## Introduction

Hematopoiesis, the continuous process of blood and immune cell production, is essential throughout an individual’s life, from embryonic development to aging [1]. This process is governed by hematopoietic stem and progenitor cells (HSPCs), which are responsible for generating oxygen-carrying red blood cells, clot-forming platelets, and various immune cells [2]. HSPCs possess an intricate network of stress-response mechanisms that enable them to respond swiftly to physiological demands while protecting themselves from damage [3].

Till and McCulloch were the first to demonstrate the existence of “self-renewing units” in the hematopoietic system, in which they showed that donor bone marrow cells could reconstitute the bone marrow and form nodules in the spleen, laying the groundwork for the later identification of hematopoietic stem cells (HSCs) [4]. In the classical model, hematopoiesis is depicted as a hierarchical process in which HSCs differentiate into various blood cell types through intermediate multipotent progenitor cells (MPPs) [5]. MPPs further differentiate into lineage-restricted progenitors, such as common lymphoid progenitors (CLPs) and common myeloid progenitors (CMPs) [6]. Recent studies have led to revisions of this classical model. Intermediate-term HSCs have been identified, with self-renewal abilities between those of long-term and short-term HSCs [7]. Additionally, MPPs have been subdivided into MPP1, MPP2, MPP3, and MPP4, each with distinct immunophenotypes and differentiation potentials [8]. Lymphoid-primed multipotent progenitors (LMPPs), identified by high expression of Flt3, preferentially generate lymphoid lineages [9]. In addition, the classical view of hematopoiesis as a stepwise process is increasingly challenged by the concept of hematopoiesis as a continuum, where differentiation occurs gradually at both cellular and molecular levels [10–12]. Single-cell RNA sequencing reveals that hematopoiesis is marked by the suppression of cell division-related genes and the activation of lineage-specific genes in a continuous manner [13].

Although HSPCs play pivotal role during hematopoiesis, researches in the last two decades highlight the indispensable role of bone marrow niche in HSPCs maintenance, hematopoietic regulation, and bone marrow reconstitution [14]. This review introduces the cell types that constitute bone marrow niche and their function in hematopoietic regulation, with a focus of emerging evidences that indicate the new function of bone marrow niche during inflammation and non-hematopoietic disease.

## Bone marrow niche in steady state

The idea of the bone marrow niche emerged from pivotal experiments conducted in the previous century. In 1978, Schofield introduced the concept of the stem cell niche, underscoring the importance of the bone marrow microenvironment in safeguarding the characteristics of stem cells. He characterized the niche as a specialized anatomical location where stem cells are sustained, their differentiation is restricted, and their population is regulated [15]. The bone marrow niche is a highly specialized microenvironment within the bone.

**Figure 1.**
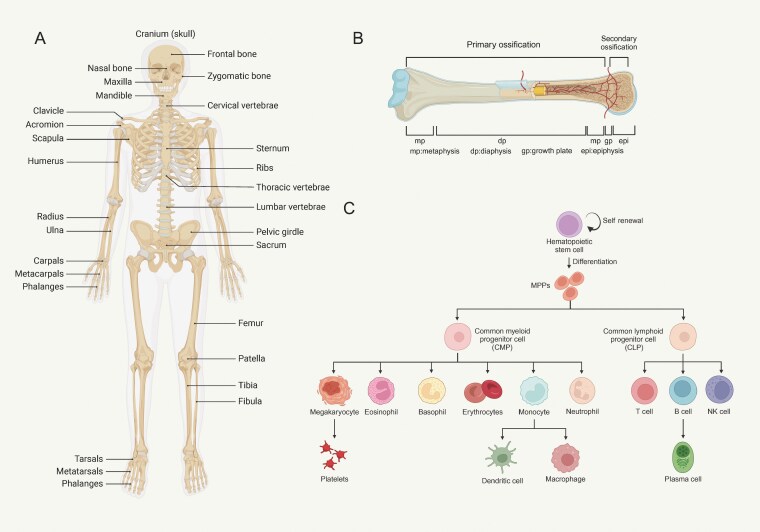
**The location and hematopoietic function of bone marrow.** (A) The spatial distribution of primary bone in human; (B) The structural division of long bone marrow; (C) The hierarchy of hematopoiesis in the bone marrow.

Based on the anatomical structure of bone, it is simply categorized into long bone and flat bone [16] (Fig. 1A). New evidence indicates potential differences of skull bone marrow (typical flat bone) and femoral bone marrow (typical long bone) in regulation of hematopoiesis [17], although their cellular components are similar, including mesenchymal stromal cells (MSCs), vascular endothelial cells (VECs), osteolineage cells, nerve, and adipocytes, all of which interact in complex ways to regulate hematopoiesis [18]. The cellular components between human and mice are similar, but their developmental emergence timing, gene expression profile and potential cell–cell interaction may not be identical [19, 20]. Further anatomical division describes the bone marrow of long bone into epiphysis, metaphysis, diaphysis and endosteum that come together to create a supportive environment for hematopoiesis (Fig. 1B and 1C). The technological advances, including advanced imaging technique and new genetically-modified animal models, have greatly enhanced our understanding of bone marrow niches.

### Mesenchymal stromal cells

**Figure 2.**
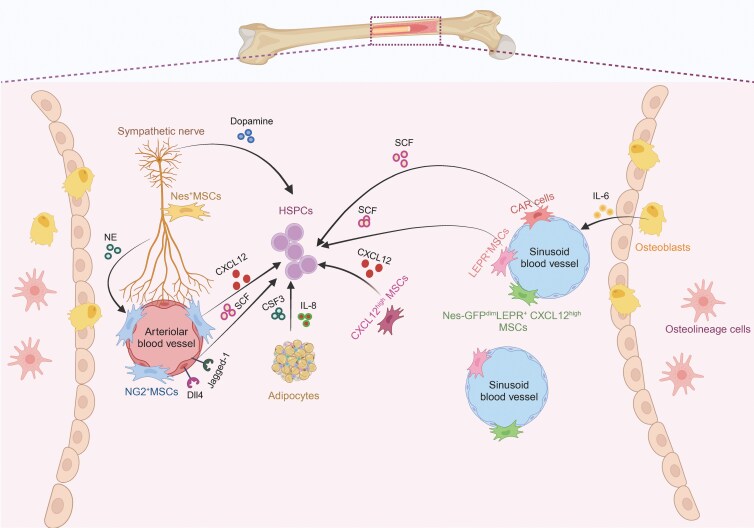
**The major components of bone marrow niche for HSPCs.** An overview cell types and major niche factor of bone marrow. Vascular endothelial cells and perivascular mesenchymal cells secrete niche factors, including SCF, CXCL12 and others, to control the maintenance, mobilization, and proliferation of HSPCs. Nerves can release noradrenaline (NE) and dopamine to indirectly or directly bind to their corresponding receptors in mesenchymal cells and HSPCs, respectively. Other niche factor could be released from adipocyte and osteolineage cells.

MSCs in the bone marrow constitute a small fraction of the total nucleated cell population, accounting for roughly 0.001%–0.01%. These cells are primarily localized in the perivascular regions, where they form close interactions with blood vessels and sympathetic nerves [21]. This strategic positioning enables MSCs as a signaling center to support lifelong blood cell production [22] (Fig. 2).

Using genetically modified mouse models, it has been demonstrated that bone marrow mesenchymal stromal cells (BM MSCs) exert specific functions by utilizing distinct molecular markers and paracrine factors, such as nestin (Nes), stem cell factor (SCF), CXCL12 [23], NG2, Prx1, and the leptin receptor (Lepr) [24]. MSCs in the bone marrow exist in distinct subpopulations, each residing in specific niches with unique phenotypes and transcriptional profiles. For instance, Nes-GFP^high^ and NG2^+^ MSCs are primarily located in the periarterial region, while Nes-GFP^mid^, Lepr^+^, and CXCL12^high^ MSCs are found in the perisinusoidal region [25]. The perisinusoidal MSCs largely overlap with CXCL12-abundant reticular (CAR) cells. The importance of CAR cells is evidenced by their ablation, which leads to a reduction in the HSCs population due to robust decrease of CXCL12 and SCF production [25]. In contrast, periarteriolar MSCs play a vital role in maintaining HSCs quiescence, as evidenced by increased distances between HSCs and arterioles following recovery from stress conditions. Nes^+^ MSCs, located near Schwann cells and sympathetic nerves, also contribute to HSCs retention within the bone marrow (BM) niche. The removal of these MSCs results in HSCs depletion in the BM and increases HSCs migration to the spleen [26, 27]. These observations highlight the functional diversity of MSC niches within different spatial location of the bone marrow.

MSCs secrete various factors, including CXCL12, SCF, angiopoietin, pleiotrophin, and others, which influence HSCs survival and expansion. The role of SCF produced by MSCs has been confirmed in experiments with LepR-Cre mice, where SCF deletion alters HSCs maintenance [28]. Deletion of CXCL12 [23] from MSCs results in significant depletion of the HSCs population, highlighting the critical role of MSC-derived factors in HSCs maintenance. Furthermore, distinct MSC subpopulations contribute differently to HSCs regulation. For example, deletion of CXCL12 in NG2^+^ MSCs affects HSCs distribution, while its deletion in Lepr^+^ MSCs has a minimal impact on HSCs [29]. Notably, although Lepr is frequently used as molecular marker of MSCs, this receptor senses systemic leptin levels to regulate the cell fate of stromal cells. Conditional deletion of LepR using Prx1-Cre does not affect body weight or normal hematopoiesis. However, bones from these knockout mice exhibited increased osteogenesis, reduced adipogenesis, and accelerated fracture healing. Leptin was shown to promote adipogenesis and inhibit osteogenesis by activating Jak2/Stat3 signaling in bone marrow stromal cells. In contrast, in Prx1-Cre Lepr knockout mice, a high-fat diet failed to increase adipogenesis and reduce osteogenesis. This suggests that Lepr influences both local osteogenesis and adipogenesis in bone marrow stromal cells, as well as systemic bone resorption [30]. MSC-derived CXCL12 was critical to influence motility of HSCs within bone marrow, therefore targeting CXCL12 and its corresponding receptor CXCR4 is indicated as an effective way to induce rapid mobilization of highly engraftable HSCs [31]. Under steady-state condition, MSCs are the primary source of pleiotrophin to maintain HSCs pool but MSC-derived pleiotrophin makes minimal contribution to hematopoietic regeneration [32]. In contrast, although the deletion of Angiopoietin-1 from MSCs promotes the recovery of bone marrow vasculature and hematopoiesis after irradiation and transplantation, it has little effect on HSCs frequency under steady-state conditions [33]. These evidences indicate that MSCs secrete multiple paracrine factors to influence HSCs and hematopoiesis at different conditions.

### Vascular endothelial cells

Together with MSC, the vascular endothelial cells (VECs) that spatially associated with MSCs constitute the vascular niche for hematopoiesis (Fig. 2). Using single cell RNA-seq (scRNA-seq), bone marrow VECs can be classified into three types: arteriolar endothelial cells (AECs), capillary endothelial cells, and sinusoidal endothelial cells (SECs) [34, 35]. Based on the spatial location and CD31/Emcn expression, the non-arterial VECs are divided into type-H and type-L blood vessel locating to metaphysis/endosteum and diaphysis, respectively [36, 37]. Despite their distinct locations and functions, most of VECs are critical for the survival and function of HSCs [38].

SECs are located along the sinusoids, which are specialized blood vessels within the BM that facilitates the exchange of cells and molecules between the marrow and the bloodstream [39]. Although SECs produce a relatively small amount of SCF and CXCL12 in adult, depletion of these microenvironmental factors in VECs results in further elimination of HSPCs [40]. SECs express relatively high level of E-selectin, Icam-1, Vcam1 as well as other adhesion molecules that involves in HSPCs adhesion to blood vessel and thereby trafficking and engraft into the bone marrow [41].

AECs promote initial engraftment of HSCs and HSPCs into embryonic bone marrow and release high level of Wnt for their expansion [20]. The extracellular matrix protein Del-1, produced by arteriolar endothelial cells and reticular cells, interacts with its receptor integrin-β3 to promote the proliferation and differentiation of HSCs, particularly toward myeloid progenitor cells [42]. AECs serve as another essential source of SCF, which is indispensable for HSPCs regeneration, especially following myeloablative treatments [43].

Both arterial and sinusoidal VECs contribute to hematopoiesis by producing Notch ligands including delta like canonical Notch ligand 4 (Dll4) and Jagged-1. Depletion of Dll4 and its downstream Rbpj lead to reduced HSCs at steady state [44]. Conditional deletion of Jagged-1 in ECs leads to HSCs exhaustion and a significant decline in hematopoiesis, underscoring its essential role in sustaining HSCs. Moreover, Jagged-1 is vital for the regeneration of hematopoiesis following myeloablation, demonstrating its importance in the recovery and maintenance of the hematopoietic system [45].

Lethal irradiation of the bone marrow results in significant damage to bone marrow VECs, and the regeneration of these cells depends on vascular endothelial growth factor receptor 2 (VEGFR2). Blocking VECs regeneration using antibodies against VE-cadherin or VEGFR2 severely inhibits hematopoietic reconstitution [46]. In addition, Apln^+^ ECs are essential for the restoration of the bone marrow vascular network and the maintenance of HSCs after bone marrow irradiation and stem cell transplantation. The removal of this endothelial cell subpopulation disrupts vascular regeneration and hematopoietic reconstitution [47].

Some microenvironmental factors, such as epidermal growth factor (EGF), can be secreted by vascular endothelial cells, especially Tie2^+^ endothelial cells, playing a crucial role in the recovery of HSCs after irradiation [48]. At the transcriptional level, the transcription factor Klf6, expressed in ECs, modulates HSCs lodgment and expansion in zebrafish via the chemokine Ccl25b [49]. Its mammalian ortholog, Ccl21, can expand hematopoietic progenitors in *ex vivo* systems, illustrating the evolutionary conservation of this regulatory mechanism.

### Osteolineage cell

Osteoblastic cells, or bone-forming cells, were initially identified as key regulators of HSPCs frequency *in vivo* [50]. However, subsequent studies have called into question whether this influence is direct (Fig. 2) [51]. It was assumed that osteoblasts played a critical role in maintaining HSCs due to their close proximity to HSCs within the bone marrow. Advanced *in vivo* imaging with validated markers (e.g. SLAM markers) or genetically labeled HSCs demonstrate that only a minority of HSCs are in direct contact with osteoblastic cells [50]. Furthermore, experiments involving osteoblast depletion—either through genetic methods like biglycan deficiency or via diphtheria toxin treatment—and those that increased osteoblast numbers through treatments like strontium, showed no significant acute effect on HSCs frequency [51]. Notably, conditional deletion of osteoblasts using diphtheria toxin led to an acute depletion of lymphoid progenitors, but had no substantial impact on HSCs [52–54].

Despite these findings, there remains a nuanced role for osteolineage cells in HSPCs regulation. Evidence for this is seen in the higher concentration of HSPCs within the trabecular-rich metaphysis and high EdU proliferating HSPCs in endosteum. This distribution may result from other bone marrow components co-localizing with bony surfaces [55]. Experiments involving conditional deletion of the transcription factor osterix, which results in osteolineage differentiation defects, showed abolished hematopoiesis in the metaphysis [56]. This indicates that the presence of mature osteolineage cells in endosteum-rich regions is crucial for sustaining hematopoiesis [57].

Osteoblasts and bony ossicles derived from mesenchymal progenitors can promote functional vascular branching by secreting VEGFA, thereby facilitating the reconstitution of HSCs and maintaining their proliferation [57, 58]. Additionally, CD105^+^Thy1^−^ progenitor cells recruit host-derived blood vessels, produce donor-derived ectopic bones through a cartilage intermediate, and generate a marrow cavity populated by host-derived long-term reconstituting HSCs (LT-HSCs) [50]. This suggests that although bone or bone-forming progenitors do not directly sustain HSCs, they can regulate the formation or maintenance of HSCs by promoting angiogenesis and branching. Furthermore, osteolineage cells can indirectly modulate the hematopoietic niche. For instance, during bone turnover, fluctuations in local ionic calcium concentrations can influence the development or engraftment of HSCs in the bone marrow via the calcium-sensing receptors on these cells [59].

Osteolineage cells also secrete various cytokines, extracellular matrix proteins, and directly transfer mitochondria to influence multiple cell types in the bone marrow. For example, activation of the parathyroid hormone receptor (PTHr) on osteoblasts leads to the expression of various regulatory molecules, including IL-6, RANKL, and Jagged1, which can affect other bone marrow cells, including blood vessels, thereby indirectly influencing HSPCs [60]. In addition, osteolineage cells transfer mitochondria to various blood cells, including myeloid cells and HSPCs. These HSPCs, in turn, transfer mitochondria to BMSCs, facilitating bone marrow niche reconstruction after irradiation and stem cell transplantation [61]. Osteoblast and osteocyte depletion impairs HSPCs mobilization into peripheral blood following granulocyte-colony stimulating factor (G-CSF) treatment, despite minimal CXCL12 expression by osteoblasts [62]. This finding suggests that while osteoblasts may not directly maintain HSPCs, they are essential for the mobilization and proper function of other progenitor cells [63].

These findings suggest that researches on osteolineage cells should shift toward examining their collaborative interactions with other microenvironmental cells in the regulation of HSPCs. The bone marrow niche, a highly complex region, comprises diverse cell types and structures, with osteolineage cells potentially exerting distinct functions based on their proximity to specific anatomical features.

### Bone marrow nerve

Another important cell type that contributes to bone marrow niche is the innervated nerve in bone marrow (Fig. 2). Our recent work shows that nerves extend and innervate embryonic BM at E17.5 at a specific anatomical structure, termed the “lesser trochanter” in femur. Genetic blockade of nerve ingression into BM does not influence HSPCs expansion during embryonic bone marrow development [64]. This is dramatically different from the diverse function of nerves in adult BM.

It has been well established that sympathetic nerves secrete noradrenaline to influence the movement of HSCs. Noradrenaline performs this function by regulating the expression of CXCL12 in MSCs, which express several noradrenaline receptors [65]. Similar to noradrenaline, recent work shows that cholinergic signal activates α7 nicotinic receptor in MSCs, which indirectly influences regenerative hematopoiesis [66–68]. In contrast to the indirect influence, recent work identifies dopamine as another nerve-derived neuromodulator that directly targets HSPCs [69]. Dopamine is necessary for the survival and proliferation of HSPCs, therefore could be targeted to enhance transplantation efficiency [69]. The direct interaction between nerves and HSCs is further supported by calcitonin gene-related peptide, which directly binds to calcitonin receptor-like receptor (CALCRL) and receptor activity modifying protein 1 (RAMP1) in HSCs [70]. γ-aminobutyric acid (GABA) in bone marrow can directly influence HSCs, which express type B receptor subunit 1 of GABA to promote transplantation [71]. VECs or MSCs derived neuronal guidance cues netrin-1 modulate dormancy and self-renewal of HSCs [72, 73]. MSCs are recently reported to release nerve growth factor to maintain nerve fibers and promote hematopoietic regeneration [74], suggesting the complicated multi-cellular crosstalk between niches and HSPCs.

### Adipocytes

The reciprocal relationship between hematopoiesis and adipose tissue within the bone marrow has been a topic of considerable interest for many years [60]. Historically, this relationship has been described by an inverse correlation: at birth, bones primarily contain red marrow, which is highly active in hematopoiesis and largely devoid of adipocytes. As individuals age, red marrow is progressively replaced by yellow marrow, characterized by an abundance of adipocytes and a marked decline in hematopoietic activity [75]. This transition led to the early conclusion that adipocytes, one of the most prevalent stromal components in adult bone marrow, function as negative regulators of hematopoiesis. Supporting this view, experimental evidence has demonstrated that mice unable to produce adipocytes or those treated with PPAR-γ receptor antagonists, which inhibit adipogenesis, exhibit significantly accelerated hematopoietic recovery following irradiation [60]. One proposed mechanism for this observation involves a positive feedback loop in which adipocytes secrete monocyte chemoattractant protein-1 (MCP-1), promoting the differentiation of mesenchymal stem cells into adipocytes while simultaneously exerting a suppressive effect on HSPCs [76].

However, recent studies have challenged this simplistic perspective, revealing a more complicated role for adipocytes within the bone marrow microenvironment, particularly under conditions of hematopoietic stress [77]. Similar to bone marrow mesenchymal stromal cells, bone marrow-derived adipocytes support the survival of HSCs *in vitro* through the secretion of key molecules, including C–X–C motif chemokine 12 (CXCL12), interleukin (IL)-8, colony-stimulating factor 3 (CSF3), and leukemia inhibitory factor (LIF) [78]. This finding suggests that adipocytes may play a supportive role in hematopoiesis, a role that had previously been overlooked.

Moreover, bone marrow adipocytes have been identified as active participants in the regeneration of HSCs and hematopoiesis following myeloablative treatments, such as irradiation or chemotherapy with 5-fluorouracil (5-FU). In these contexts, adipocytes, along with their precursor cells, including a subpopulation of leptin receptor-positive cells produce SCF, has been shown to be particularly crucial for hematopoietic regeneration. Conditional deletion of SCF in adipocytes significantly impairs hematopoietic recovery, highlighting the importance of these cells in supporting HSCs survival and expansion during hematopoietic stress [79].

Notably, the role of adipocytes in hematopoiesis extends beyond simple support or inhibition; they act as key modulators, particularly during periods of hematopoietic stress. For instance, studies have shown that PPARγ knockout mice, which lack adipocytes, exhibit severe extramedullary hematopoiesis, a condition where hematopoiesis occurs outside the bone marrow, often in the spleen or liver [60]. This phenomenon may be related to dysregulation of the CXCL12/CXCR4 axis, suggesting that adipocytes play a critical role in the retention and mobilization of HSCs within the bone marrow. These evidences indicate that while adipocytes were once viewed solely as negative regulators of hematopoiesis, recent data presents a more complex and dynamic role.

### Small extracellular vesicles

In addition to the traditional crosstalk via ligand–receptor interactions, small extracellular vesicles are recently emerged as a new way to mediate the crosstalk between bone marrow niche and HSPC. Small extracellular vesicles (SEV) are membrane-bound particles, typically ranging from 30 to 150 nm, that are produced by various cell types in both physiological and pathological conditions. In addition to the traditional ligand receptor crosstalk, these vesicles play a vital role in intercellular communication by transferring molecular contents, such as proteins, lipids, RNA, and even DNA, between cells [80].

Recent studies have highlighted the role of SEV in the interaction between HSPCs and the bone marrow niche, particularly in hematological malignancies such as leukemia. In one study, the *Vps33b* gene was knocked out in multiple cell types using seven cell-type-specific mouse Cre lines. These lines included endothelial cells (Cdh5^+^ or Tie2^+^), bone marrow perivascular cells (Lepr^+^), osteoprogenitor cells (Osx^+^), megakaryocytes (Pf4^+^), and spleen stromal cells (Tcf21^+^). The results showed that blocking SEV secretion from VECs significantly delayed leukemia progression, while no effect was observed when SEV production was inhibited in perivascular cells, megakaryocytes, or spleen stromal cells. Furthermore, blocking SEV production in endothelial cells did not impact normal hematopoiesis. VEC-derived SEV contained high levels of ANGPTL2, a protein that accelerates leukemia progression by binding to the LILRB2 receptor. The release of ANGPTL2-enriched SEVs from VECs was regulated by VPS33B. These findings suggest that bone marrow niche-specific SEV play a significant role in leukemia progression [81].

In additional *in vitro* and murine xenograft studies, evidence demonstrated that AML exosomes downregulate key retention factors such as SCF and CXCL12 in stromal cells, thus promoting the mobilization of HSPCs from the bone marrow. Moreover, the trafficking of exosomes directly impacted HSPCs by downregulating CXCR4 and cKit expression, influencing key signaling pathways, including c-Myb, Cebp-β, and Hoxa-9 [82]. SEV have also been shown to carry miR-126, which supports the quiescence, self-renewal, and engraftment of leukemia cells. The downregulation of miR-126 in leukemia cells was linked to the phosphorylation of Sprouty-related EVH1-domain-containing 1 (SPRED1) by BCR-ABL, which inhibited the RAN–exportin-5–RCC1 complex responsible for miRNA maturation. Additionally, VECs in the bone marrow were found to supply miR-126 to leukemia cells, as demonstrated in mouse models where miR-126 was conditionally knocked out in VECs [83].

Together, these findings, along with additional studies [84–87], support the potential of small extracellular vesicles as a promising target for investigating the crosstalk between HSPCs and the bone marrow niche. Targeting these interactions could offer new therapeutic strategies to alleviate leukemia progression.

## Bone marrow niche adaptation to inflammation and emergent hematopoiesis

**Figure 3.**
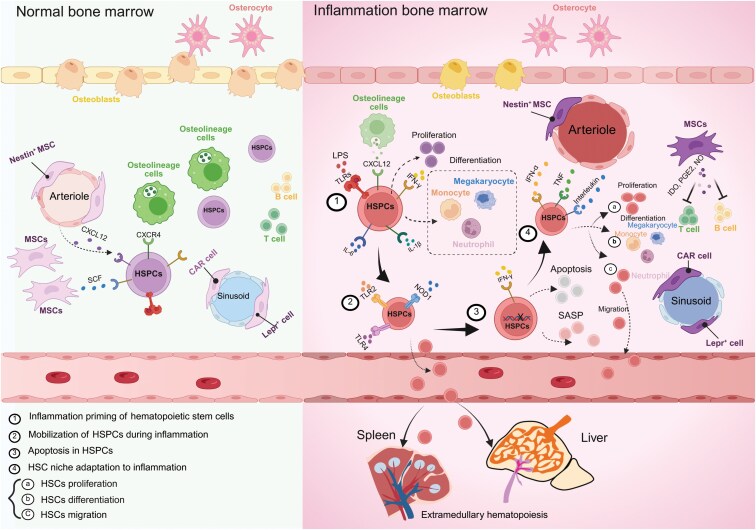
**Comparison of normal and inflammatory bone marrow.** Inflammation induces priming, mobilization and apoptosis of hematopoietic stem and progenitor cells. The hematopoietic change is coupled with adaptation of bone marrow niche.

The hematopoietic stress prompts HSCs to exit their quiescent state, leading to proliferation and lineage differentiation according to the specific demands of the stressor. During systemic inflammation or infection, the bone marrow must rapidly produce mature myeloid cells, particularly neutrophils and monocytes, in a process termed emergency hematopoiesis (Fig. 3) [88]. HSPCs are equipped with pattern-recognition receptors, such as Toll-like receptors (TLRs), that directly detect pathogen-derived molecules circulating in the bone marrow [89]. Activation of TLRs on HSPCs enhances their proliferation and drives myeloid cell production, ensuring a swift replenishment of these critical immune cells during infection [89].

HSPCs are responsive to cytokines released by immune cells at sites of inflammation, as well as by cells within the bone marrow microenvironment. Pro-inflammatory cytokines, such as interleukin-1 (IL-1), interleukin-6 (IL-6), tumor necrosis factor (TNF) [90], and interferon (IFN) [91], directly promote HSPCs proliferation and drive their differentiation toward the myeloid lineage. However, prolonged exposure to these cytokines can severely impair HSCs function. Therefore, it is crucial to understand how bone marrow niche cells and the cytokines they secrete regulate HSPCs behavior in inflammatory contexts, as this knowledge is essential for precisely controlling the inflammatory response and facilitating the restoration of tissue homeostasis.

### Bone marrow niche adaptation to inflammation

#### Mesenchymal stromal cells

Inflammation has a profound impact on MSCs function and their ability to support HSPCs. Upon exposure to inflammatory signals, MSCs undergo significant changes, including the upregulation of immunosuppressive molecules that modulate the immune response within the bone marrow. These immunosuppressive mediators, such as indoleamine 2,3-dioxygenase (IDO), prostaglandin E2 (PGE2), and nitric oxide (NO), inhibit the proliferation of T cells, B cells, and natural killer (NK) cells, helping to create an immunosuppressive niche that protects HSPCs from excessive immune activation during inflammation [22].

Another interesting mechanism by which MSCs modulate immune responses during inflammation is through the transfer of mitochondria to CD4^+^ T cells. This transfer induces the differentiation of these T cells into regulatory T cells (Tregs), which are crucial for restraining inflammatory responses and maintaining immune homeostasis. This mitochondrial transfer highlights the diverse ways in which MSCs contribute to the regulation of the bone marrow niche during inflammation [92].

However, chronic inflammation can also impair the supportive function of MSCs, leading to remodeling of the HSPCs niche. For example, viral infections, such as those caused by lymphocytic choriomeningitis virus (LCMV), can trigger IFN-γ signals that remodel the structural network of the bone marrow niche [93]. This remodeling suppresses the function of CAR cells, a subset of MSCs, reducing their ability to secrete HSPC-supportive molecules. The result is a compromised niche that is less capable of maintaining HSPCs quiescence and function, leading to impaired hematopoiesis [93].

#### Vascular endothelial cells

During inflammation, the function and structure of endothelial cells in the bone marrow are significantly altered. For example, lipopolysaccharide (LPS) or viral mimics like polyinosinic-polycytidylic acid (pIpC), increased sinusoid diameter and reduced VECs cellularity [94]. This vascular remodeling alters the production of key hematopoietic factors like SCF and Notch ligands (DLL1/4, JAG2). Additionally, upregulation of IFN-α during viral infections further stimulates VECs through enhanced VEGF signaling, promoting VECs proliferation and altering the vascular niche [95]. Chronic inflammation, particularly when driven by endothelial mitogen-activated protein kinase (MAPK) activation, can further impair vascular integrity and HSPCs activities [96, 97]. In mouse models of chronic inflammation, the impaired vascular integrity and reduced HSPCs function are associated with increased cellular hypoxia and ROS generation [98]. These conditions exacerbate the inflammatory niche and further disrupt the bone marrow niche. However, inhibition of NF-κB in these models has been shown to restore vascular integrity and improve HSCs survival, mobilization, and reconstitution, highlighting the potential therapeutic value of targeting endothelial inflammation in the bone marrow niche [99].

Danger-associated molecular patterns released by damaged or stressed cells, along with pathogen-associated molecular patterns, can initiate an inflammatory response [100]. Bone marrow endothelial cells (BMECs) detect these molecules via pattern recognition receptors and Toll-like receptors, leading to the activation of NF-κB and the subsequent expression of various pro-inflammatory cytokines, including G-CSF, granulocyte-macrophage colony-stimulating factor (GM-CSF), IFN, IFN, IL-1, TNF, and IL-6. This cytokine cascade facilitates the proliferation, migration, and differentiation of HSPCs [101].

These responses are particularly evident in conditions such as bacteremia induced by severe *Escherichia coli* infection, where G-CSF and GM-CSF function in both paracrine and autocrine modes. Specifically, GM-CSF triggers a positive feedback loop that amplifies cytokine production, thereby exacerbating the inflammatory response [102]. Concurrently, BMECs downregulate CXCL12 and SCF, which are crucial for maintaining the HSCs pool during acute and chronic inflammation. For instance, in diabetes mellitus, where patients are at heightened risk for atherosclerosis, BMECs chronically downregulate HSC-protective factors CXCL12 and Ang-1 due to disrupted epidermal growth factor receptor (EGFR) signaling [103].

In response to pro-inflammatory stimuli such as LPS and TNFα, BMECs increase the expression of various activation markers, including Jagged-2 (Jag2), which plays a key role in supporting HSCs proliferation [104]. Inflammatory conditions also lead to the development of hypoxic regions within the bone marrow, which subsequently induces the upregulation of vascular endothelial growth factor (VEGF) and its receptor VEGFR2 by both endothelial cells and HSCs. This coordinated response promotes enhanced angiogenesis, further facilitating the bone marrow’s adaptation to inflammatory challenges [105].

#### Osteolineage cell

During inflammation, the function and survival of osteoblasts are significantly impacted, leading to alterations in the bone marrow niche that can disrupt HSCs maintenance. For instance, during bacterial infections such as those caused by *Staphylococcus aureus*, osteoblasts are suppressed, leading to bone loss and osteomyelitis [106]. This suppression is mediated through the action of G-CSF, which increases in response to infection. G-CSF has been shown to alter the morphology and gene expression of osteoblasts, primarily by modulating adrenergic signals from the sympathetic nervous system. This modulation not only reduces osteoblast activity but also affects the production of key HSPC-supportive factors [40].

Despite the suppression of osteoblasts, G-CSF alone does not trigger HPSCs mobilization when osteoblasts are depleted. This indicates that osteoblasts are indispensable for HSCs mobilization, likely through their collaborative function with other cell types in the niche. One such collaborative cell type is the osteal macrophage [107], which supports osteoblast function and is crucial for maintaining the endosteal niche. Osteal macrophage help sustain HSPCs by promoting the production of maintenance factors, such as CXCL12, SCF, and Ang-1 [107, 108]. However, during G-CSF treatment, osteal macrophage are depleted, leading to a disruption of the endosteal niche and a subsequent reduction in HSPCs maintenance. This depletion also impairs erythroid recovery, especially under conditions of myeloablative stress, suggesting that osteal macrophages (osteomacs) might also interact with megakaryocytes to indirectly regulate HSCs under inflammatory conditions [65].

#### Bone marrow nerve

In the peri-nervous system including bone marrow, the sympathetic and parasympathetic nervous systems exhibit distinct responses to inflammation. A localized immune response depends on the regulated recruitment of leukocytes to sites of inflammation. Adrenergic signals have been shown to play a role in the regulation of leukocyte trafficking during inflammation [109]. This process is partly mediated by the sympathetic nervous system, which influences the expression of adhesion molecules on venular endothelial cells [110]. In areas with higher sympathetic nerve density, mice exhibit increased sensitivity to inflammatory stimuli. In contrast, cholinergic stimulation has been demonstrated to suppress the expression of endothelial cell adhesion molecules via the nicotinic acetylcholine receptor alpha7 subunit (α7nAChR) [111], suggesting that the sympathetic and parasympathetic nervous systems have opposing roles in the inflammatory response.

The parasympathetic nervous system detects inflammation through interleukin-1β (IL-1β) receptors located on afferent vagus nerve fibers and chemosensory cells in the surrounding paraganglia [112]. This marks an early phase of the inflammatory reflex, in which the parasympathetic nervous system counters systemic inflammation by releasing acetylcholine. The increased release of acetylcholine inhibits endotoxin-induced pro-inflammatory cytokine production, while sparing the release of anti-inflammatory cytokines via α7nAChR on macrophages [113].

Thus, both branches of the autonomic nervous system, the parasympathetic and sympathetic systems, contribute to the regulation of immune responses. Recent studies have further demonstrated that electro-acupuncture, through activation of the sensory sciatic nerve, can reduce inflammation in experimental sepsis models, likely via dopamine production in the adrenal glands. This suggests a novel anti-inflammatory mechanism involving catecholamine modulation through the sciatic and vagus nerves. Additionally, ischemic stroke has been shown to activate HSCs through increased sympathetic tone, promoting a shift toward myeloid hematopoiesis and an enhanced bone marrow output of inflammatory monocytes and neutrophils [114].

#### Adipocytes

In healthy adults, approximately 10% of the adipose tissue in the bone marrow plays a key role in regulating whole-body energy metabolism and modulating both local and systemic inflammatory responses. Recent reviews have highlighted the involvement of bone marrow adipose tissue in these processes [115]. Bone marrow adipocytes are known to express and secrete adiponectin and leptin, which are essential in metabolic regulation. Studies in both mice and humans have demonstrated that bone marrow adipocytes express and release higher levels of adiponectin compared to white adipose tissue [116]. Additionally, changes in bone marrow adipocyte mass are associated with fluctuations in serum adiponectin levels, especially following caloric restriction [116].

It is important to note that the bone marrow serves as the primary source of immune cells for other adipose depots and metabolic organs during obesity. Therefore, it can be concluded that cytokine production by bone marrow adipocytes primarily affects bone marrow dynamics. Their contribution to systemic inflammation in obesity is likely indirect, facilitated by the production and release of immune cells.

### Inflammation-induced HSPC dynamics

#### Inflammation priming of HSPCs

During inflammation, the demand for myeloid cells, such as neutrophils and macrophages, significantly increases. These cells are vital to the immune response, serving as the first line of defense against pathogens and playing key roles in phagocytosis, the release of inflammatory mediators, and the activation of other immune cells [117]. To satisfy this heightened demand, the process of emergency hematopoiesis is initiated. This rapid response mechanism involves the activation of HSPCs, prompting them to exit their quiescent state, re-enter the cell cycle, and undergo proliferation and differentiation into myeloid lineages [118](Fig. 3).

HSPCs, similar to mature immune cells and committed myeloid progenitors in the bone marrow, can directly respond to acute infections or chronic inflammatory conditions [68]. This adaptability of HSPCs is intricately regulated by a combination of cell-intrinsic mechanisms (including transcriptional, epigenetic, and metabolic pathways) and bone marrow niche derived cell-extrinsic factors (such as soluble growth factors, cytokines, microbial ligands, and adhesive interactions) [67]. HSPCs exhibit a heightened sensitivity to external stimuli, which is greater than traditionally understood, due to their expression of receptors for microbial products, such as TLRs, as well as receptors for inflammatory cytokines and growth factors like IL-1β, IL-6, macrophage colony-stimulating factor (M-CSF), and type I and II IFNs [119]. The levels of these signaling molecules significantly increase during systemic infection, allowing HSPCs to directly or indirectly detect infectious or inflammatory challenges. Although such responses by HSPCs can facilitate the elimination of infections, chronic activation may impair HSPCs function, leading to exhaustion and potentially contributing to the persistence of inflammatory diseases.

The identification that HSPCs express TLRs and that TLR activation, via the adaptor protein MyD88, drives myeloid differentiation has significantly advanced our understanding of the role of HSPCs in immune defense against pathogens [89]. Subsequent investigations have elucidated both indirect cytokine-mediated and direct effects of TLR ligands on HSPCs. For example, upon TLR stimulation, short-term HSCs and multipotent progenitors generate substantial cytokines, predominantly through the activation of the NF-κB transcription factor [119]. Among these cytokines, interleukin-6 (IL-6) plays a critical paracrine role in promoting the proliferation and myeloid differentiation of HSPCs [119]. Systemic administration of LPS has been shown to enhance HSCs proliferation [120]. Furthermore, high-dose LPS exposure leads to increased bone marrow cell apoptosis and HSCs dysfunction, despite initial proliferative responses, while low-dose LPS exposure induces HSCs expansion accompanied by a myeloid-biased differentiation, a process mediated through an intrinsic TLR4-dependent mechanism, as evidenced by bone marrow chimera studies in TLR4-deficient mice [121]. Notably, prolonged exposure to low-dose endotoxins disrupts the quiescent state of HSCs and impairs their functional capacity, as demonstrated by reduced repopulation efficiency in serial transplantation experiments [122].

Colony-stimulating factor (CSF) serves as a critical regulator of granulopoiesis, operating under both homeostatic and emergency inflammatory conditions. It achieves this by influencing the expression of transcription factors and receptors that are specific to the myeloid lineage, thereby modulating growth factor signaling pathways essential for myeloid differentiation [117, 123]. Additional growth factors, such as M-CSF and GM-CSF, are crucial regulators of emergency myelopoiesis [117]. In the context of emergency granulopoiesis, G-CSF facilitates proliferative signaling and promotes lineage commitment. This process predominantly targets CMPs and granulocyte-macrophage progenitors (GMPs), driving their expansion into distinct progenitor clusters that subsequently differentiate into granulocytes [124, 125]. Beyond its effects on myeloid progenitor differentiation, G-CSF can also stimulate HSPCs proliferation [126]. Moreover, G-CSF promotes HSPCs mobilization from the BM indirectly by acting on monocytes within the bone marrow niche. The generation of monocytes in the BM is dependent on the receptor CSF1R, which binds M-CSF. M-CSF directly acts on hematopoietic and myeloid progenitor cells, inducing their differentiation into monocytes [127]. M-CSF can establish a myeloid lineage identity in individual HSCs, independent of HSCs survival or proliferation, by inducing a PU.1-dependent molecular signature. This M-CSF-dependent mechanism of myelopoiesis may provide protection against opportunistic infections following HSCs transplantation [128].

#### Mobilization of HSPCs during inflammation

In addition to driving the activation and proliferation of HSPCs, inflammation also triggers their mobilization from the bone marrow into the peripheral blood. This process is essential for extramedullary hematopoiesis, which occurs outside the bone marrow, and for the replenishment of immune cells at sites of infection or injury. The mobilization of HSPCs is a tightly regulated process that ensures that these cells are available where they are most needed during an immune response [129].

The mobilization of HSPCs is facilitated by various inflammatory signals and involves the activation of specific receptors on HSPCs, such as nucleotide-binding oligomerization domain-containing protein 1 (NOD1), TLR2, and TLR4 [130–133]. These receptors detect inflammatory cues and trigger signaling pathways that enable HSPCs to detach from their bone marrow niche and enter the circulation [134]. This detachment is often accompanied by changes in the expression of adhesion molecules and chemokines that normally anchor HSPCs within the bone marrow. For instance, G-CSF, a cytokine commonly used in clinical settings to mobilize HSCs for transplantation, reduces the expression of stromal cell-derived factor-1 (SDF-1/CXCL12) and vascular cell adhesion molecule 1 (VCAM-1), both of which are critical for HSPCs retention in the bone marrow niche [24].

Once mobilized into the peripheral blood, HSPCs can travel to peripheral tissues, such as the spleen, where they contribute to extramedullary hematopoiesis. This process is particularly important during severe or chronic infections, where the bone marrow alone may not be sufficient to meet the body’s hematopoietic demands. The mobilization of HSPCs also facilitates their recruitment to sites of tissue damage or inflammation, where they can support tissue repair and regeneration [65].

Interestingly, during infection, HSPCs that remain in the bone marrow exhibit increased migratory behavior, moving away from their original bone marrow niche [135]. This movement suggests that inflammation can disrupt the normal interactions between HSPCs and their niche, forcing the cells to seek out more supportive environments where they can restore their fitness. This dynamic behavior highlights the adaptability of HSPCs during inflammation, as they continuously adjust their location and activity in response to changing environmental conditions [136].

However, the mobilization of HSPCs is not without risks. The detachment of HSPCs from their bone marrow niche can expose them to a more hostile environment in the peripheral blood, where they may encounter higher levels of oxidative stress, inflammatory cytokines, and other factors that can damage the cells or impair their function [137]. Additionally, the repeated mobilization of HSPCs during chronic inflammation may deplete the bone marrow’s HSCs pool, leading to long-term impairments in hematopoietic function [138].

### Mechanism of bone marrow niche factors on the regulation of emergent hematopoiesis

#### Effect of IFN

Type 1 and type 2 IFNs, particularly IFN-α and IFN-γ, play critical roles in the adaptation of HSPCs to inflammatory conditions [70]. Type 1 IFNs, such as IFN-α, can drive the proliferation of dormant HSCs through a signaling pathway that involves the IFN-α/β receptor (IFNAR) and STAT1 [139]. However, prolonged exposure to IFN-α has detrimental effects, impairing the repopulation capacity of HSCs. The transcription factor IRF2, which negatively regulates type 1 IFN signaling, serves to protect quiescent HSCs from exhaustion induced by type 1 IFN-driven proliferation [140]. The functional decline and attrition of HSCs under sustained type 1 IFN signaling are associated with the induction of DNA damage in HSCs as they re-enter the cell cycle, coupled with increased mitochondrial activity and reactive oxygen species (ROS) production. Although IFN-α initiates HSCs proliferation, this response is often brief due to a temporary reduction in the expression of genes that enforce quiescence, such as *p27*, *p57*, *Foxo1*, *Foxo3a*, and *Pten* [141]. The re-establishment of quiescence in HSCs can shield them from the pro-apoptotic effects of IFN-α [142]. Additionally, HSCs may deploy other protective mechanisms to counteract IFN-α-induced dysfunction. For instance, signaling induced by all-trans retinoic acid has been shown to protect dormant HSCs from polyI:C-induced replicative stress.

Furthermore, the cyclic GMP–AMP (cGAMP) synthase cGAS, which senses intracellular pathogen-derived DNA, predominantly from viruses, plays a role in this context. Upon detecting DNA, cGAS generates cGAMP, which, in conjunction with the adapter protein STING, stimulates type 1 IFN production [143]. Interestingly, a circular RNA, cia-cGAS, which antagonizes cGAS activity, is highly enriched in the nucleus of long-term HSCs. This RNA helps maintain HSCs dormancy by protecting them from cGAS-mediated, type 1 IFN-induced exhaustion. The complexity of type 1 IFN effects on the bone marrow is further exemplified by findings that acute IFN-α-mediated inflammation can impact the bone marrow niche, particularly endothelial cells, altering vascularity and vessel permeability. Additionally, type 1 IFN can promote proliferation and post-transcriptional protein synthesis in a primed subpopulation of stem cell-like megakaryocyte-committed progenitors within the HSCs population, leading to rapid platelet production during acute inflammation [144].

IFN-γ, a type 2 IFN, also plays a significant role in modulating HSCs maintenance and proliferation. Using an *in vivo Mycobacterium avium* infection model, it was found that a higher proportion of long-term repopulating HSCs proliferate during infection, and this response requires IFN-γ signaling but not IFN-α signaling. IFN-γ-deficient mice show a reduced proliferative rate, suggesting that baseline IFN-γ levels regulate HSC activity [141]. However, the effects of IFN-γ on HSCs are highly context-dependent. IFN-γ impairs the maintenance of HSCs by directly reducing their proliferative capacity and hindering their recovery during viral infections. This effect is mediated through the induction of SOCS1 expression in HSCs, which inhibits TPO-induced STAT5 phosphorylation, subsequently disrupting the regulation of key cell-cycle genes [145]. Similarly, intravenous administration of the mycobacterial Bacille Calmette-Guérin (BCG) vaccine triggers IFN-γ-dependent expansion of HSPCs and promotes their differentiation toward the myeloid lineage via a myelopoiesis-related transcriptional program [146].

#### Effect of IL-1β

IL-1β, a key mediator of innate immunity, directly influences HSPCs by promoting their proliferation and differentiation into myeloid lineages, primarily through the activation of the transcription factor PU.1 [147]. In experimental models, administration of IL-1β to mice leads to an increased population of myeloid-biased HSPCs, whereas bone marrow recovery following chemotherapeutic injury is notably impaired in mice lacking the IL-1 receptor. Chronic exposure to IL-1β, however, reduces the self-renewal capacity of HSCs, although this adverse effect can be reversed upon the cessation of IL-1β signaling [148].

IL-1 also plays a role in persistent age-related inflammation and inducible trained immunity. For example, β-glucan induces IL-1-dependent adaptations in bone marrow hematopoietic progenitors, promoting the expansion of specific HSC subsets and myelopoiesis [149]. Additionally, older mice produce more 1L-1α and 1L-1β in steady-state bone marrow compared to younger SPF mice. In older WT mice, antibiotic-mediated suppression of the microbiota or pharmacological blockade of IL-1 signaling similarly reverses the myeloid-biased output of their HSC populations [150]. These findings highlight the critical role of IL-1 signaling in regulating both hematopoietic function and immune responses.

Interestingly, short-term or low-dose administration of IL-1α or IL-1β has been shown to prevent chemotherapy-induced myelosuppression and protect against sepsis in neutropenic mice induced by cyclophosphamide. This suggests that the timing and dosage of IL-1 exposure are critical factors in determining its beneficial or harmful effects on HSCs. While IL-1 can facilitate the restoration of myelopoiesis, prolonged IL-1-mediated inflammation is detrimental to HSCs function, highlighting the importance of regulating IL-1 signaling in therapeutic contexts [96].

#### Effect of other interleukins

Additionally, IL-3, which is predominantly produced by immune cells, is minimally detectable in healthy or non-inflammatory tissues [151, 152]. However, it is significantly upregulated at sites of inflammation, underscoring its role as a crucial regulator of the immune response. For instance, the expansion of HSPCs, as well as myeloid progenitors, is significantly impaired in IL-3-deficient mice during sepsis. Additionally, elevated plasma IL-3 levels are associated with increased mortality in human sepsis cases [153].

IL-6 is another critical regulator of HSPCs proliferation and differentiation, particularly during emergency myelopoiesis. IL-6 can originate from HSPCs themselves following TLR stimulation or from bone marrow mesenchymal stem cells (BM-MSCs) in response to IFN-γ released by cytotoxic T cells. Moreover, IL-6, through signaling pathways involving SHP2 and STAT3, supports the survival of TET2-deficient HSPCs and myeloid cells [154]. Given that TET2 mutations are associated with clonal hematopoiesis and an elevated risk of leukemia, these findings suggest that inflammation may contribute to the survival and expansion of preleukemic HSPCs.

IL-27, a member of the IL-6/IL-12 cytokine family, influences HSPCs by promoting emergency myelopoiesis. For example, IL-27 exerts antitumor effects by enhancing HSC expansion and differentiation into antitumorigenic M1 macrophages in tumor-bearing mice [155]. Furthermore, aging-related inflammation accelerates HSC aging via the TNFα→ERK→ETS1→IL27Ra pathway. Deletion of IL27Ra mitigates HSC functional decline, myeloid bias, and the inhibitory effects of TNFα on HSCs [156]. These observations underscore the complex interplay between cytokines and HSPCs in regulating hematopoiesis during inflammatory conditions.

#### Effect of TNF

Recent studies have revealed the critical role of TNFα in promoting HSC survival and differentiation, particularly under the influence of inflammatory signals. Specifically, TNFα has been shown to activate a robust and specific p65/NF-κB-dependent gene program, primarily preventing necroptosis rather than conventional apoptosis. This is significant because necroptosis is a more inflammatory form of cell death, which could exacerbate tissue damage and hinder regeneration. By preventing necroptosis, TNFα ensures the survival of HSCs during periods of inflammation, allowing them to contribute to blood regeneration [157]. In addition to its role in survival, TNFα has also been demonstrated to induce myeloid differentiation in HSCs through PU.1, preparing them for the production of myeloid cells, which are crucial for immune responses, especially in the context of infection or injury [158].

The role of TNFα in HSC regeneration extends beyond acute inflammatory responses. In fact, TNFα also plays a role in chronic inflammation and aging [159]. For example, in elderly individuals, the regenerative capacity of HSCs diminishes, and the inflammatory environment, particularly elevated levels of TNFα, may exacerbate the decline in HSC function. Nevertheless, the continued presence of TNFα signaling helps to maintain HSC function, although this is accompanied by an increase in myeloid differentiation and a reduction in lymphoid output [156]. This shift in lineage production is a hallmark of aging in the hematopoietic system and may contribute to increased susceptibility to infections and hematopoietic malignancies in the elderly. Furthermore, TNFα also plays a key role in HSC transplantation, as research has shown that TNFα derived from granulocytes can be transferred to BMECs, promoting vascular regeneration and enhancing HSC survival [160].

## Hematopoiesis and niche dysfunction in non-hematopoietic disease

Various diseases can significantly disrupt the normal functioning of the bone marrow niche and hematopoiesis [99], which may affect the BM through inflammatory processes, leading to changes in the niche that can alter HSPCs behavior and overall hematopoietic output [40].

Here, we elucidate the impact of several organ-specific diseases on bone marrow niche and hematopoiesis, including cardiovascular diseases, chronic kidney disease, metabolic disorders, and autoimmune diseases.

### Cardiometabolic disease

Cardiovascular diseases (CVDs) are a group of disorders affecting the heart and blood vessels, which include conditions such as atherosclerosis, hypertension, and heart failure. Recent studies have highlighted the intricate interplay between the cardiovascular system and bone marrow, revealing how cardiovascular pathologies influence bone marrow hematopoiesis and the hematopoietic niche.

Following myocardial infarction (MI), the sudden loss of cardiac tissue triggers a robust inflammatory response, which extends beyond the heart to systemic organs, including the bone marrow [161]. The acute inflammation associated with MI stimulates emergency hematopoiesis in the bone marrow, characterized by the rapid activation and expansion of HSPCs. This process leads to the increased production of neutrophils and monocytes, which are mobilized from the bone marrow to the site of injury to participate in the inflammatory response and tissue repair. However, this hyperactivation of hematopoiesis can also lead to long-term alterations in the bone marrow niche, including the depletion of HSCs and the exhaustion of the bone marrow regeneration capacity, potentially contributing to chronic inflammatory states and recurrent cardiovascular events [162].

**Figure 4.**
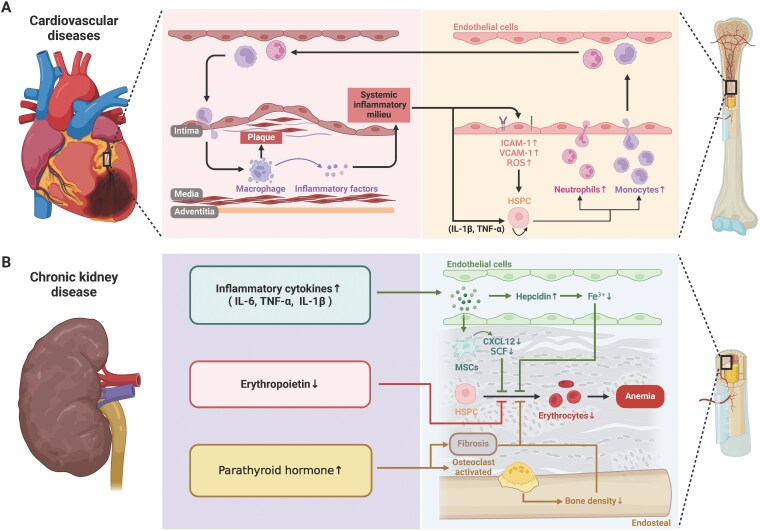
**Cardiovascular disease and chronic kidney disease influences hematopoiesis and bone marrow niche.** (A) Cardiovascular disease influences monocytes and neutrophils production together with change of adhesion molecules in vascular endothelial cells to regulate their trans-endothelial migration. The myeloid cell participates in the plaque formation in cardiovascular disease. (B) CKD is coupled with increased inflammatory cytokine production, reduced red blood cell generation and release of parathyroid hormone.

Atherosclerosis is characterized by the accumulation of lipids and inflammatory cells in the arterial walls, leading to plaque formation. Recent studies have identified a phenomenon known as “trained immunity,” where bone marrow-derived myeloid cells, particularly monocytes and macrophages, exhibit a heightened and sustained inflammatory response following exposure to atherogenic stimuli [163]. This trained immunity is driven by epigenetic reprogramming of HSPCs in the bone marrow, which are exposed to systemic inflammatory mediators, such as IL-1β and TNFα. These cytokines activate signaling pathways that enhance the proliferation and myelopoiesis of HSPCs, leading to an increased production of pro-inflammatory monocytes that contribute to the progression of atherosclerotic lesions [164](Fig. 4A).

For the alterations of niche cells, the systemic inflammatory milieu promotes the upregulation of adhesion molecules such as VCAM-1 and ICAM-1 [165] on BMECs, which influences HSC-niche interactions and increases HSCs mobilization. Additionally, the production of ROS in response to cardiovascular stressors can damage endothelial cells, leading to increased vascular permeability and the disruption of the vascular niche, ultimately impairing HSCs maintenance and function [166]. Such dysfunction leads to abnormal expansion of myeloid cells, particularly monocytes and neutrophils. Once these cells are produced in the bone marrow and enter the circulatory system, they tend to exhibit enhanced pro-inflammatory and chemotactic properties, making them more likely to accumulate in damaged cardiovascular areas. For instance, the accumulation of myeloid cells within arterial walls can exacerbate the formation and instability of atherosclerotic plaques. The rupture of these plaques can lead to severe cardiovascular events such as myocardial infarction or stroke. Furthermore, these myeloid cells exacerbate the systemic inflammatory state by releasing various inflammatory factors, thereby accelerating the progression of cardiovascular diseases [167].

Therefore, there exists a complex feedback loop between bone marrow endothelial dysfunction and the expansion of myeloid cells, which not only drives the onset and progression of cardiovascular diseases but may also serve as a primary driver of their continued deterioration. Increasing evidence suggests that the bone marrow niche plays a central role in this process. By understanding and intervening in the dysfunction of BMECs, it might be possible to discover new therapeutic approaches to block the abnormal expansion of myeloid cells, thereby reducing the inflammatory burden on the cardiovascular system and slowing or even reversing the disease process.

### Chronic kidney disease

Chronic kidney disease (CKD) is a long-term condition characterized by a gradual loss of kidney function, often leading to end-stage renal disease (ESRD) and requiring dialysis or kidney transplantation. CKD is associated with a wide range of systemic complications, including anemia, cardiovascular disease, and bone mineral disorders. These complications are closely linked to disruptions in bone marrow hematopoiesis and the bone marrow niche [168].

One of the most prominent hematologic complications of CKD is anemia, which is primarily caused by impaired erythropoiesis in the bone marrow [169](Fig. 4B). The kidneys play a crucial role in erythropoiesis by producing erythropoietin (EPO), a hormone that stimulates the production of red blood cells (RBCs) in the bone marrow [170]. As kidney function declines in CKD, the production of EPO decreases, leading to reduced stimulation of erythropoiesis and the development of anemia.

Iron deficiency is another common factor contributing to anemia in CKD. The regulation of iron metabolism is closely linked to erythropoiesis, and CKD can disrupt this balance in several ways [171]. Inflammatory cytokines, which are often elevated in CKD, stimulate the production of hepcidin, a hormone that inhibits intestinal iron absorption and iron release from macrophages [172]. Elevated hepcidin levels in CKD reduce the availability of iron for erythropoiesis, leading to iron-restricted erythropoiesis and contributing to the severity of anemia [173]. Furthermore, the chronic inflammation associated with CKD can lead to functional iron deficiency, where iron is sequestered in macrophages and not available for RBC production, despite adequate iron stores [174].

CKD is often accompanied by mineral and bone disorders, collectively known as CKD–MBD (chronic kidney disease–mineral and bone disorder) [175]. These disorders include alterations in calcium, phosphate, parathyroid hormone (PTH), and vitamin D metabolism, which can significantly affect bone health and the endosteal niche in the bone marrow [176, 177]. Elevated PTH levels in CKD can stimulate osteoclast activity, leading to increased bone resorption and reduced bone density [178]. This disruption of the bone architecture can alter the endosteal niche, impacting the ability of osteoblasts to support HSCs maintenance and function [179]. Additionally, changes in bone mineralization and increased bone turnover in CKD–MBD can lead to a less stable niche environment, further impairing hematopoiesis. CKD can also lead to fibrosis within the bone marrow, characterized by the accumulation of extracellular matrix components and the replacement of normal bone marrow tissue with fibrous tissue. Fibrosis in the bone marrow disrupts the normal architecture of the hematopoietic niche, reducing the available space for hematopoiesis and impairing the function of HSCs. The presence of fibrosis in the bone marrow is associated with poor hematopoietic function and contributes to the progression of anemia and other hematologic complications in CKD [180].

CKD is associated with chronic systemic inflammation, which significantly impacts the bone marrow niche. The inflammatory milieu in CKD is characterized by elevated levels of pro-inflammatory cytokines, such as IL-6, TNFα, and IL-1β. These cytokines can alter the function of bone marrow stromal cells, including MSCs and endothelial cells, leading to a disrupted niche environment [14, 181]. For example, inflammation-induced changes in MSCs can result in altered secretion of key hematopoietic factors, such as CXCL12 and SCF, which are essential for maintaining HSCs quiescence and supporting erythropoiesis. Additionally, chronic inflammation can promote the senescence of MSCs, reducing their regenerative capacity and impairing their ability to support hematopoiesis [182, 183]. Additionally, the altered bone marrow niche in CKD may contribute to the increased risk of clonal hematopoiesis, where mutated HSCs gain a competitive advantage and expand, potentially leading to hematologic malignancies or other complications.

CKD significantly impacts bone marrow hematopoiesis and the hematopoietic microenvironment, leading to the development of anemia, impaired erythropoiesis, and other hematologic complications. The chronic inflammation associated with CKD disrupts the bone marrow niche, altering the function of MSCs, endothelial cells, and osteoblasts, and contributing to fibrosis and bone mineral disorders. These changes not only impair hematopoiesis but also exacerbate the progression of CKD and its associated comorbidities, particularly cardiovascular disease. Understanding the interplay between CKD and bone marrow hematopoiesis is crucial for developing targeted therapies that can improve hematologic outcomes and reduce the burden of CKD-related complications [168].

### Metabolic disorders

**Figure 5.**
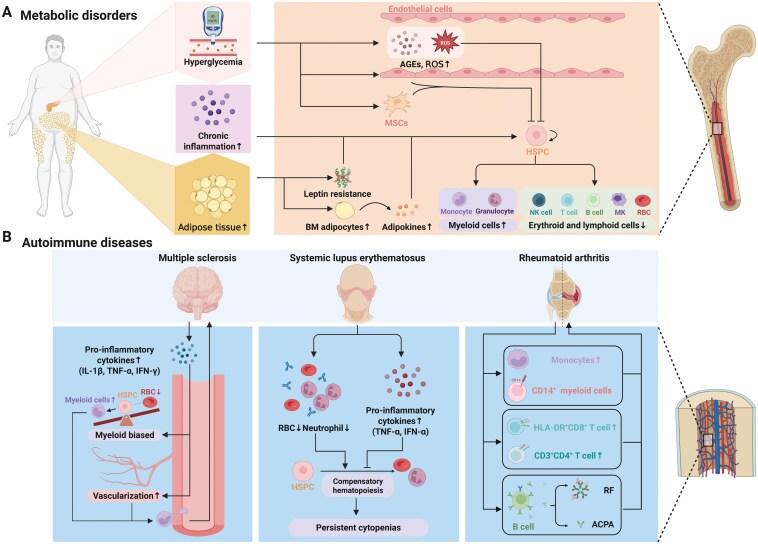
**Metabolic disorder and autoimmune disease influences hematopoiesis and bone marrow niche.** (A) Metabolic disorders associated with hematopoietic disorders include hyperglycemia, chronic inflammation and adipose tissue increase. These disorders influence release of niche factor from adipocyte leading to myeloid cell overproduction and decrease of red blood cell and lymphoid cells. (B) Autoimmune diseases associated with hematopoietic disorders include MS, lupus erythematosus, and RA. These disorders have their unique influence to hematopoiesis and bone marrow niche.

Metabolic disorders, including diabetes mellitus and obesity, are chronic conditions that significantly affect various physiological systems by chronic low-grade inflammation, insulin resistance, and dysregulated metabolism, all of which have profound effects on bone marrow hematopoiesis and the bone marrow niche [184](Fig. 5A).

#### Diabetes mellitus

Diabetes mellitus, encompassing both Type 1 and Type 2 diabetes, is primarily characterized by persistent high blood sugar levels. In Type 1 diabetes, this is due to insulin deficiency, while in Type 2, it results from insulin resistance [185]. Both forms of diabetes induce substantial systemic complications, including those affecting the bone marrow [186]. Chronic hyperglycemia leads to the formation of advanced glycation end-products (AGEs) and increased oxidative stress, which impair the ability of HSCs to proliferate, differentiate, and self-renew effectively [187]. Moreover, hyperglycemia disrupts the bone marrow niche, particularly by altering the function of mesenchymal stem cells and endothelial cells that are crucial for maintaining a healthy hematopoietic environment. The cumulative effect is a significant reduction in the bone marrow’s capacity to sustain normal blood cell production, leading to various hematologic complications. For example, in BM aspirates of type 2 diabetic patients, CD34^+^ cell number are reduced [188]. This condition is further complicated by chronic inflammation, a common feature of insulin resistance, which exacerbates the adverse effects on the bone marrow microenvironment. The result is a skewed production of myeloid cells at the expense of erythroid and lymphoid cells, increasing the risk of anemia and immune system dysfunction in individuals with diabetes [189].

#### Obesity

Obesity, defined by excessive fat accumulation, is closely associated with a state of chronic low-grade inflammation, termed “metaflammation,” which significantly alters the bone marrow’s microenvironment and disrupts normal hematopoietic processes. The expansion of adipose tissue in obesity leads to the increased production of pro-inflammatory cytokines, such as TNFα, IL-6, and MCP-1. This ongoing inflammation spreads to the BM, where it interferes with the normal function of HSCs and other hematopoietic cells [190]. Additionally, the infiltration of adipocytes into the BM, known as “bone marrow adiposity,” exacerbates hematopoietic dysregulation [191]. Bone marrow adipocytes release adipokines and other factors that inhibit the proliferation and differentiation of HSCs, reducing the bone marrow’s overall hematopoietic capacity and increasing the risk of anemia, leukopenia, and other blood-related abnormalities in obese individuals [79]. Leptin, a hormone produced by adipose tissue, plays a crucial role in regulating both energy balance and hematopoiesis, which is known to promote HSCs proliferation and myelopoiesis. However, in obesity, the body often becomes resistant to leptin, diminishing its effectiveness, leading to a dysregulated hematopoietic process, causing an imbalance in blood cell production [99]. This condition is further aggravated by the increased production of pro-inflammatory cytokines associated with leptin resistance, which perpetuates the inflammatory environment in the BM and worsens hematopoietic dysregulation.

### Autoimmune diseases

Autoimmune diseases are characterized by the immune system’s aberrant attack on the body’s own tissues, leading to chronic inflammation and tissue damage. These diseases can significantly affect bone marrow hematopoiesis and the bone marrow niche, resulting in hematologic abnormalities and further complicating disease management [192](Fig. 5B).

#### Multiple sclerosis

Multiple sclerosis (MS) is a chronic autoimmune disorder primarily affecting the central nervous system (CNS), where it causes demyelination and neurodegeneration [193]. In the beginning stages, the disease typically follows a relapsing remitting (RRMS) course that is marked by recurrent episodes of inflammation in the central nervous system (CNS), and pathologically marked by inflammatory infiltrates that are abundant in macrophages and T and B cells [194, 195]. Increasing axonal and neuronal loss, a steady decrease in inflammation, and a gradual buildup of impairment are the hallmarks of the subsequent secondary progressive MS (SPMS) phase. While MS is generally considered a neurological disease, it also has far-reaching systemic effects, including significant impacts on the BM and the process of hematopoiesis [196]. Haematopoietic stem cell transplantation (HSCT) used in MS for over two decades [197]. It is mostly used as an immunomodulatory and anti-inflammatory therapy for multiple sclerosis. By using high-dose chemotherapy to deplete the autoreactive immunologic memory and then profoundly regenerating a new and diversified immune system—a process known as “immune reset”—aHSCT is carried out with the goal of reconstituting, and ideally reconditioning, the immune system toward a self-tolerant state [198–201]. Numerous post-transplant mechanistic investigations in MS have demonstrated that new thymic production of T cells emerges after aHSCT, and that the T-cell repertoire, especially of CD4 T cells, may be nearly entirely replenished and more diverse [202]. While this treatment can potentially reconstitute the immune system and lead to long-term remission, it also carries risks such as myelosuppression—a reduction in BM activity that can lead to decreased blood cell production—and other complications related to BM recovery [203]. Therefore, understanding how MS affects the bone marrow niche and hematopoiesis is essential for optimizing treatment strategies and improving outcomes for patients with MS.

#### Systemic lupus erythematosus

Systemic lupus erythematosus (SLE) is a multisystem autoimmune disease characterized by the production of autoantibodies and immune complexes, leading to widespread inflammation and tissue damage [204]. Autoimmune cytopenias, including autoimmune hemolytic anemia, immune thrombocytopenia, and autoimmune neutropenia, are common in SLE [205]. In SLE, most cells participating in the pathogenesis of SLE originate from BM HSPCs, indicating that immune cells in SLE could be traced back to HSPCs [206].

Transcriptomic analysis of HSPCs derived from lupus-affected mice revealed a pronounced myeloid signature characterized by an increased prevalence of common myeloid progenitors. In individuals diagnosed with SLE exhibiting severe manifestations of the disease, hematopoietic progenitor cells (CD34) showed increased rates of proliferation, differentiation, and transcriptional activation of cytokines and chemokines that promote myelopoiesis, thereby reflecting findings observed in murine studies [207]. Additionally, myelofibrosis has documented in patients with SLE. Sporadic observations have indicated that BM fibrosis could be included within the spectrum of SLE. The correlation between the management of the disease and the amelioration of pancytopenia offers indirect support for a potential causal link between SLE and BM fibrosis. Furthermore, autoimmune myelofibrosis may exhibit a positive response to immunosuppressive therapies, leading to a reduction in fibrosis and the restoration of normal BM function [208].

#### Rheumatoid arthritis

Rheumatoid arthritis (RA) is a chronic autoimmune disorder that primarily affects the joints but also manifests systemically, including hematologic abnormalities [209]. One key aspect of RA pathogenesis involves increased local production of inflammatory cytokines, which, along with altered cell–cell interactions, contribute to morphological, immunophenotypic, and functional abnormalities in bone marrow cells [210]. Both myeloid and lymphoid lineage cells are affected in RA. For instance, the total number of mononuclear cells is significantly increased in RA patients compared to healthy individuals [211]. Additionally, RA patients exhibit accelerated maturation of CD14^+^ myeloid cells from BM progenitors and enhanced spontaneous production of these cells *in vitro*, indicating potential changes in the BM’s regenerative capacity [212, 213].

In addition to these alterations in the myeloid compartment, evidence suggests that the BM microenvironment in RA may support aberrant B- and T-cell immune responses. Specifically, B cells from the BM of RA patients produce disease-associated antibodies, such as IgM anti-citrullinated peptide antibodies (ACPAs) and various forms of rheumatoid factor (RF) [214]. Furthermore, the iliac BM of RA patients shows an increased number of HLA-DR^+^CD8^+^ and recently activated CD3^+^CD4^+^ T lymphocytes, highlighting the role of T-cell dysregulation in the disease process [215, 216]. Hematopoietic stem cell transplantation (HSCT) has emerged as a promising treatment for various autoimmune diseases, including RA, particularly in cases that are refractory to conventional therapies. In RA patients, HSCT has demonstrated the potential to induce remission, though the duration of this remission typically lasts up to two years [217].

## Conclusion and perspective

Hematopoiesis is a fine-tuned process essential for the lifelong production of blood cells, ensuring that the body’s physiological needs are met. The regulation of hematopoiesis during development and steady-state maintenance has been extensively investigated. This regulation becomes even more critical under stress condition, such as myeloablative injury and leukemia. In this review, we highlight how hematopoiesis is controlled during inflammation, infection as well as multiple non-hematopoietic disease, which is overlooked by most of other reviews.

Additionally, this hematopoietic stress condition is accompanied by disruption of the bone marrow niche. The bone marrow niche dysfunction can lead to an overproduction of inflammatory cytokines, causing HSPCs to exit quiescence and enter continuous proliferation, eventually leading to HSCs exhaustion or reduced level of growth factors that is necessary for survival maintenance of HSPCs. Therefore, maintaining a healthy bone marrow niche is crucial for proper hematopoiesis. Further investigation of the relationships between bone marrow niche dysfunction and non-hematopoietic disease could be vital for developing new therapeutic strategies to preserve or restore niche function, achieving a precise control of the hematopoietic condition of our body and ultimately supporting effective disease management.
